# Retromammary Schwannoma Mimicking a Breast Tumor in a Patient with Neurofibromatosis Type 1: A Case Report Demonstrating Surgical Avoidance Using Contrast-Enhanced Ultrasound–Informed Biopsy

**DOI:** 10.70352/scrj.cr.26-0169

**Published:** 2026-09-08

**Authors:** Hirokazu Yamazaki, Shinsaku Kanazawa, Kazuko Ozawa, Hajime Yuasa, Hitoshi Tsuda

**Affiliations:** 1 Department of Breast Surgery, Community Hospital Koga, Yaizu, Shizuoka, Japan; 2 Department of Breast Surgery, Iwase General Hospital, Sukagawa, Fukushima, Japan; 3 Department of Dermatology, Community Hospital Koga, Yaizu, Shizuoka, Japan; 4 Tenryu Koseikai Medical Clinic, Hamamatsu, Shizuoka, Japan; 5 Department of Diagnostic Pathology, Seikei-kai Chiba Medical Center, Chiba, Chiba, Japan

**Keywords:** schwannoma, retromammary space, neurofibromatosis type 1, contrast-enhanced US, vacuum-assisted biopsy

## Abstract

**INTRODUCTION:**

Retromammary schwannomas are rare peripheral nerve sheath tumors that may clinically and radiologically mimic breast lesions, occasionally leading to unnecessary surgical intervention.

**CASE PRESENTATION:**

We report a young Japanese woman with previously undiagnosed neurofibromatosis type 1 who presented with breast pain. US revealed a mobile, well-circumscribed hypoechoic mass located in the retromammary space. Contrast-enhanced US (CEUS) using a second-generation contrast agent demonstrated a characteristic dynamic target sign, and MRI showed concordant findings. Vacuum-assisted biopsy performed with reference to the CEUS target-like heterogeneous enhancement pattern confirmed a benign schwannoma.

**CONCLUSIONS:**

Dynamic US assessment combined with CEUS-informed biopsy target selection may improve diagnostic confidence for retromammary lesions and help avoid unnecessary surgical resection.

## Abbreviations

CEUS
: contrast-enhanced US
MPNST
: malignant peripheral nerve sheath tumor
NF1
: neurofibromatosis type 1
PNST
: peripheral nerve sheath tumor
VAB
: vacuum-assisted biopsy

## INTRODUCTION

Schwannomas, also known as neurilemmomas, are benign tumors arising from Schwann cells of the peripheral nerve sheath. They most frequently occur in the head, neck, and extremities, whereas their occurrence in the breast region—including the retromammary space—is rare. When such lesions arise adjacent to or beneath the breast parenchyma, they can clinically and radiologically mimic breast tumors, making accurate imaging differentiation essential for appropriate diagnostic and management decisions.^1,2)^

NF1 is an autosomal dominant genetic disorder characterized by multiple neurofibromas, café-au-lait macules, and skeletal abnormalities. Revised diagnostic criteria published in 2021 incorporate both clinical and genetic features to improve diagnostic accuracy.^3)^ Although NF1 is associated with an increased risk of PNSTs, schwannomas are relatively uncommon in NF1 compared with neurofibromas, and cases in which a chest-wall schwannoma presents as a palpable breast mass are rarely documented.^4,5)^

Here, we report a retromammary schwannoma in a young Japanese woman with previously undiagnosed NF1. Imaging revealed a well-circumscribed hypoechoic lesion located in the retromammary space, and dynamic US assessment confirmed that the lesion did not arise from the breast parenchyma. This case highlights 2 key points: first, the importance of careful US evaluation of lesion mobility and posterior depth in distinguishing retromammary lesions from true intramammary tumors; and second, the potential of CEUS-informed biopsy target selection based on intratumoral perfusion patterns as an approach to optimize biopsy site selection. Accurate differentiation of retromammary lesions is essential not only to establish a correct diagnosis but also to guide appropriate surgical decision-making and avoid unnecessary surgical excision.

## CASE PRESENTATION

A Japanese woman in her 20s presented to the Department of Breast Surgery at Community Hospital Koga with a complaint of left-sided breast pain. She had no significant medical or reproductive history and no formally diagnosed family history of NF1, although her mother exhibited similar cutaneous findings.

**Fig. 1:**
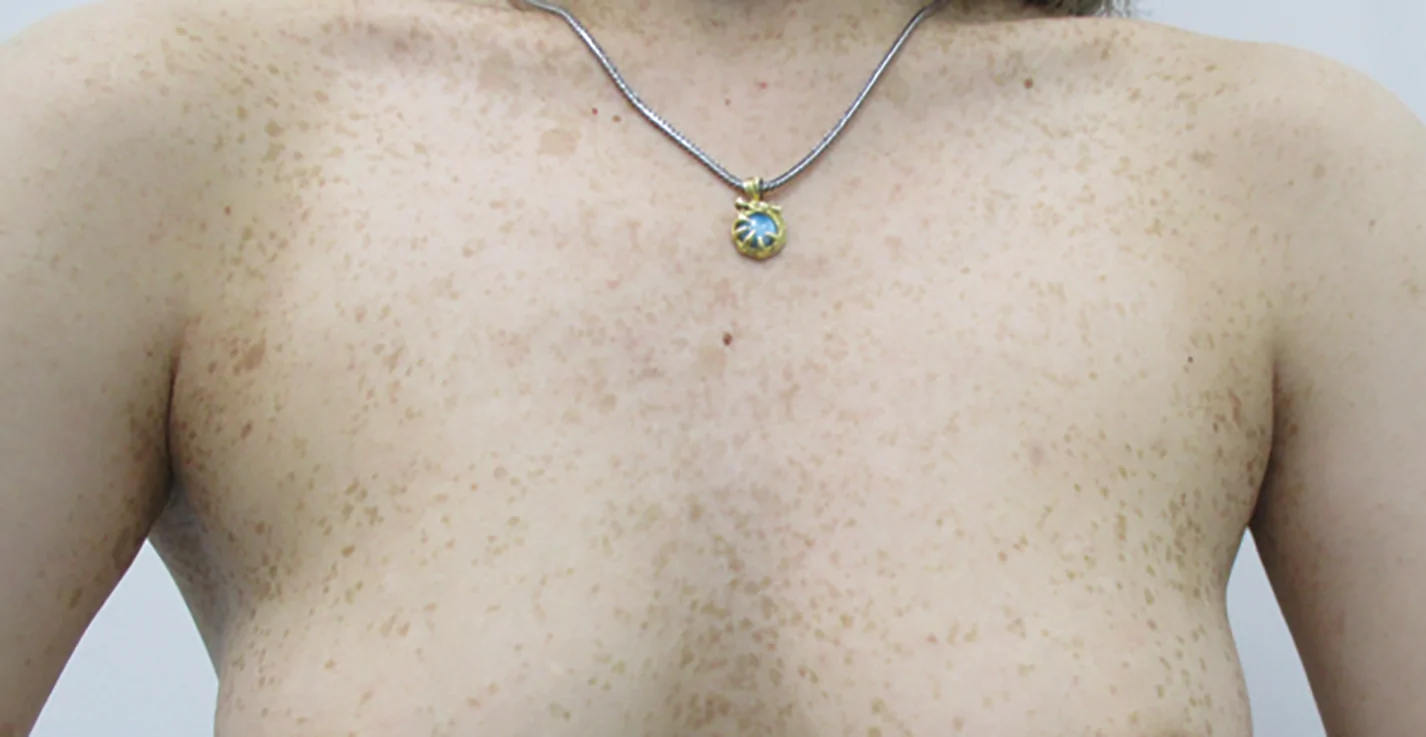
Clinical appearance of the anterior chest showing multiple café-au-lait macules and axillary freckling, consistent with the diagnostic features of NF1. NF1, neurofibromatosis type 1

Physical examination revealed multiple café-au-lait macules on the trunk and axillary freckling (**Fig. 1**). A dermatologic consultation confirmed the diagnosis of NF1 according to the revised 2021 consensus criteria, based on the presence of ≥6 café-au-lait macules and bilateral axillary freckling.^3)^

US examinations were performed using a high-frequency 18-MHz linear transducer on an Aplio i800 US system (Canon Medical Systems, Tochigi, Japan). For CEUS, a second-generation US contrast agent (perflubutane microbubbles) was administered intravenously as a bolus injection, followed by a saline flush, and real-time low–mechanical index imaging was used to assess lesion perfusion dynamics according to the manufacturer’s protocol.

**Fig. 2:**
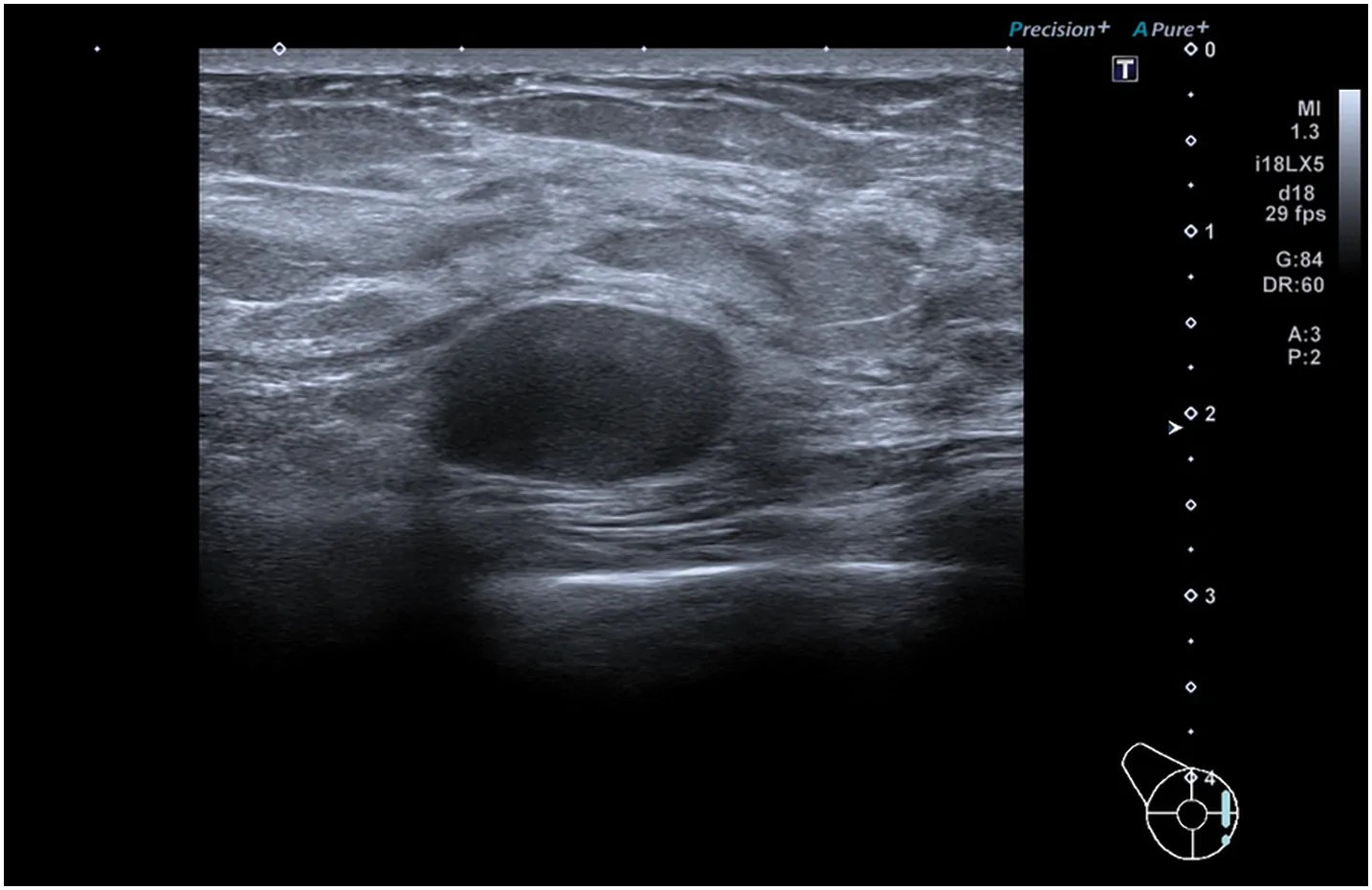
A well-circumscribed, oval, hypoechoic mass measuring 34 × 11 × 14 mm is seen in the retromammary region of the right breast, demonstrating posterior acoustic enhancement.

Bilateral breast ultrasonography was performed. While no abnormality was detected in the symptomatic left breast, a well-circumscribed, oval, hypoechoic mass measuring 34 × 11 × 14 mm was incidentally identified in the right retromammary region, showing posterior acoustic enhancement (**Fig. 2**). The internal echotexture of the mass was relatively homogeneous, and no fluid component was identified within the lesion. The lesion was located in the retromammary space, immediately superficial to the pectoralis major muscle, and shifted cranially during right arm elevation. This displacement with arm elevation indicated that the lesion moved independently of the overlying breast tissue, supporting a retromammary origin (**Supplementary Video S1**). Color Doppler imaging demonstrated minimal peripheral vascularity.

Perflubutane microbubbles were injected intravenously as a bolus, followed immediately by a saline flush. The timer displayed on the US system was started at the beginning of the saline flush, and all enhancement times reported in this study refer to this timer. CEUS demonstrated a target-like heterogeneous enhancement pattern within the lesion approximately 10–15 seconds after the start of the saline flush, as measured using the timer displayed on the US system. The lesion showed relative central hypoenhancement with surrounding enhancement, followed by gradual and mild enhancement of the remaining lesion during the late phase. This dynamic pattern produced a well-defined halo-like appearance, forming a characteristic “target sign” (**Supplementary Video S2**). The enhancement gradually diminished but persisted with slow, sustained contrast retention during the late phase, findings suggestive of a benign neurogenic tumor.

**Fig. 3:**
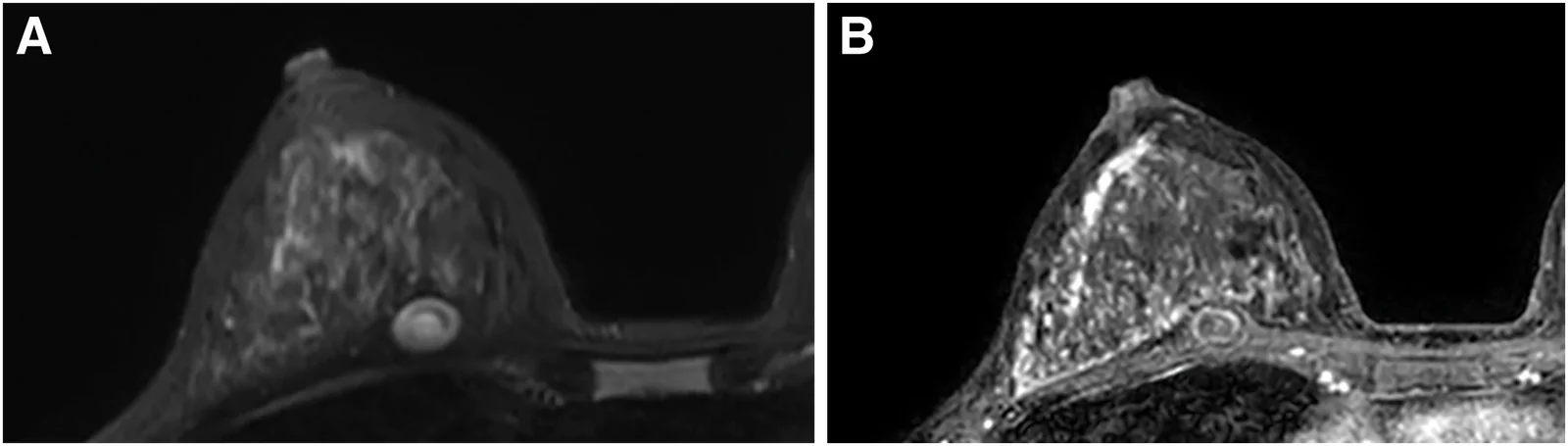
(**A**) T2-weighted fat-suppressed axial MRI demonstrating a “target sign” appearance with high peripheral and low central signal intensity. (**B**) Dynamic contrast-enhanced MRI of the right breast lesion demonstrating peripheral rim enhancement. This enhancement pattern was interpreted in conjunction with the T2-weighted target sign and other imaging findings but was not specific for schwannoma.

MRI revealed a similar target sign on fat-suppressed T2-weighted axial images, characterized by high peripheral and low central signal intensity (**Fig. 3A**). Dynamic contrast-enhanced MRI demonstrated peripheral rim enhancement (**Fig. 3B**), which prompted consideration of differential diagnoses such as invasive ductal carcinoma with central necrosis or breast abscess. However, B-mode US showed relatively homogeneous internal echotexture without fluid components, and CEUS revealed no evidence of intralesional necrosis. Taken together, the MRI and CEUS findings favored a benign neurogenic tumor such as schwannoma.

**Fig. 4:**
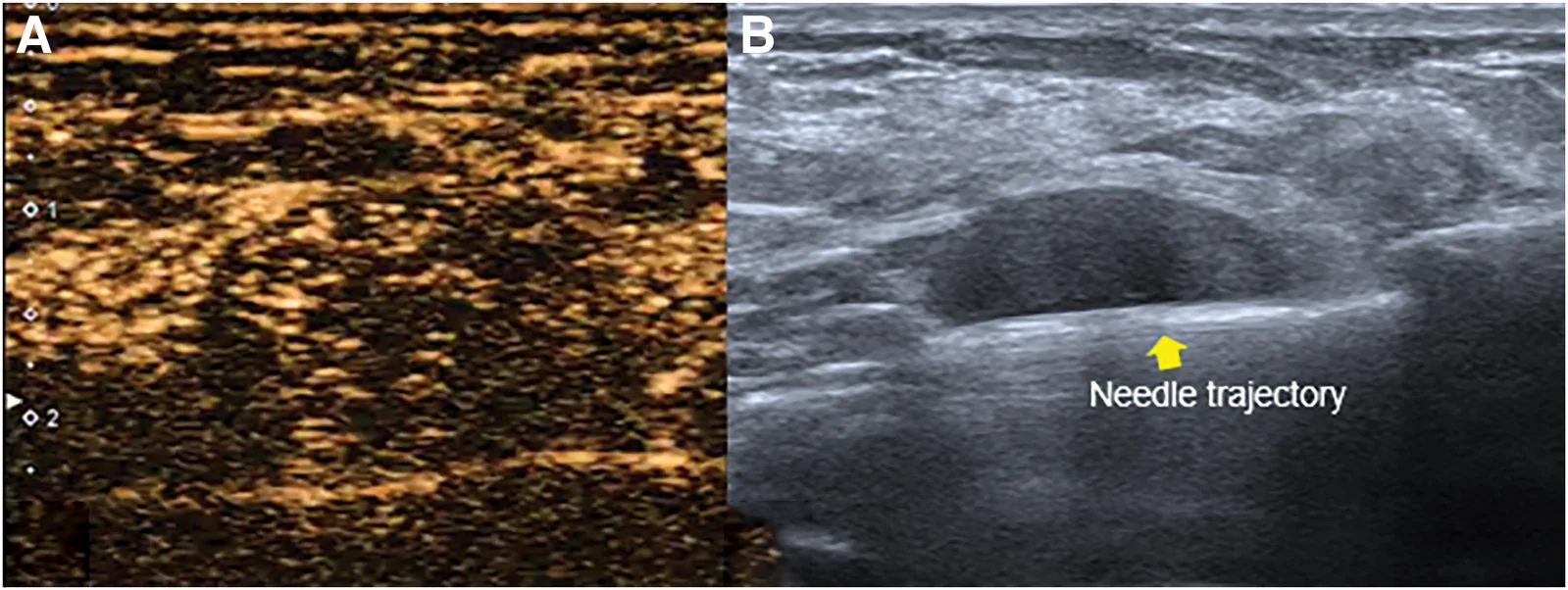
Representative images showing the CEUS enhancement pattern and biopsy trajectory. (**A**) CEUS image demonstrating a target-like heterogeneous enhancement pattern with relative central hypoenhancement and surrounding enhancement. (**B**) B-mode US image obtained during VAB, showing the needle trajectory directed through the central portion of the lesion under real-time guidance. Although the biopsy target was selected with reference to the CEUS findings, exact spatial correspondence between the CEUS enhancement pattern and the histological field could not be established. CEUS, contrast-enhanced US; VAB, vacuum-assisted biopsy

**Fig. 5:**
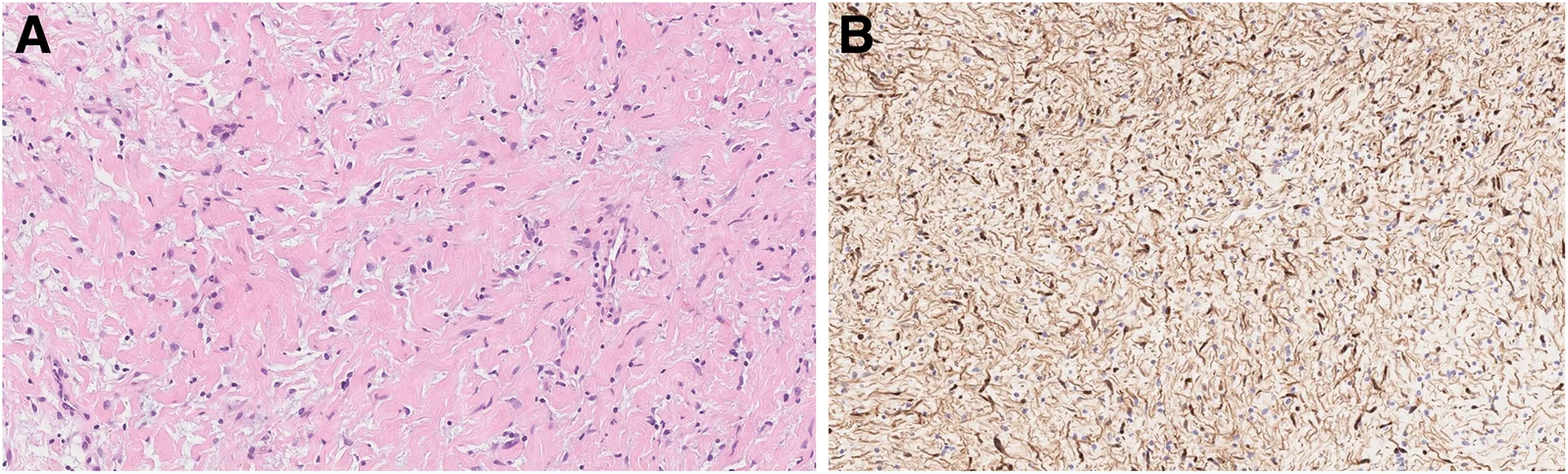
(**A**) Histopathological image showing loose Antoni B architecture with wavy spindle cells without nuclear atypia or mitoses. Lymphocytic infiltration is also seen. (**B**) Immunohistochemical staining demonstrating diffuse, strong positivity for S-100 protein, confirming schwannoma.

Based on the CEUS findings, the biopsy was directed toward the central portion of the lesion, which was included in the target-like heterogeneous enhancement pattern identified on CEUS (**Fig. 4A**). US-guided VAB was performed without complication. After CEUS assessment, VAB was performed under real-time B-mode US guidance with reference to the target-like heterogeneous enhancement pattern. Local anesthesia with 10 mL of 1% lidocaine was infiltrated into the skin, subcutaneous tissue, and around the lesion before biopsy. To minimize the risk of pneumothorax, the biopsy was performed under continuous real-time US guidance with the entire needle visualized throughout the procedure. Because the lesion was located adjacent to the chest wall, the patient position and skin entry site were adjusted to achieve a needle trajectory as parallel as possible to the chest wall, thereby avoiding a steep needle angle toward the pleura (**Fig. 4B**). US-guided VAB was performed using a 12-gauge vacuum-assisted breast biopsy device (Hologic, Marlborough, MA, USA), and 2 core specimens were obtained without complication. Histopathological analysis showed loose Antoni B architecture, and no definite Antoni A component was identified in the biopsy specimen (**Fig. 5A**). Regional differences were observed within the Antoni B component, with collagen-rich stroma in the outer to intermediate portions and more myxoid stroma in the central portion. Immunohistochemical staining showed strong and diffuse positivity for S-100 protein (**Fig. 5B**), confirming a diagnosis of benign schwannoma.

Because the lesion mimicked an intramammary tumor, surgical excision was initially considered. However, the concordant imaging and histopathological findings supported a benign neurogenic tumor, and conservative management with clinical observation was therefore selected, avoiding unnecessary surgical resection. At 12-month follow-up, no interval growth or new lesions were observed on imaging, and no additional neurogenic tumors were identified elsewhere in the body.

## DISCUSSION

This case highlights the importance of accurate preoperative localization of deep retromammary lesions, as misinterpretation as intramammary tumors may lead to unnecessary surgical excision. Accurate preoperative localization together with functional imaging assessment directly influenced surgical decision-making in this patient. Schwannomas are benign PNSTs that are rarely found in the breast and may mimic common benign entities such as fibroadenomas or phyllodes tumors, particularly when located deep near the chest wall.^1,2)^ Schwannomas arising in the retromammary space are rare, and retromammary or anterior chest-wall schwannomas presenting as breast tumors have been reported only sporadically.^4,5)^ In the present case, the lesion initially appeared similar to a benign intramammary tumor due to its oval, well-circumscribed morphology. However, dynamic US assessment later demonstrated that it originated from the retromammary space.

On US, the mass was oval, hypoechoic, and showed posterior acoustic enhancement with minimal internal vascularity—features consistent with benign schwannoma morphology.^6)^ A key diagnostic feature in this case was lesion mobility observed during dynamic US maneuvers, in which the mass shifted cranially with arm elevation. This mobility suggested a subfascial or neurogenic origin rather than attachment to the glandular parenchyma. Prior work has emphasized that deep breast lesions should prompt attention to posterior fat, obtuse chest-wall angles, and pectoralis proximity when considering non-glandular origin.^7,8)^

CEUS demonstrated a target-like heterogeneous enhancement pattern characterized by relative central hypoenhancement with surrounding enhancement. This pattern produced a stable “target-like” appearance and closely matched the target sign described by Yang and Cui in schwannomas, further supporting the diagnosis of a PNST.^6)^ MRI provided complementary evidence, demonstrating a classic target sign on T2-weighted imaging and peripheral rim enhancement. Rim enhancement on breast MRI is not specific to schwannomas and may also be observed in invasive ductal carcinoma with central necrosis, fat necrosis, organized hematoma, or abscess formations. However, in our case, B-mode US showed relatively homogeneous internal echotexture without fluid components, and CEUS revealed no intralesional necrosis. These imaging findings made necrotic or inflammatory breast lesions less likely and supported the interpretation of benign PNSTs, although definitive subtype diagnosis still required histopathological confirmation.^9)^

Although schwannomas are typically benign, special consideration is warranted in patients with NF1. Farid et al.^10)^ and Evans et al.^11)^ reported that patients with NF1 have an estimated 8%–13% lifetime risk of developing MPNSTs. Malignant change may occur focally within an otherwise benign-appearing lesion; therefore, careful selection of the biopsy target is important to reduce sampling error. In our patient, the absence of atypia or mitoses in the region sampled with reference to the CEUS enhancement pattern further supported a benign diagnosis despite the underlying NF1.

In this context, CEUS enables real-time visualization of intratumoral perfusion dynamics and may help identify regions with distinct perfusion characteristics. Sampling such regions may reduce the risk of non-representative biopsy in selected PNSTs, although CEUS enhancement should not be interpreted as direct evidence of increased cellularity or Antoni A architecture. In the present case, VAB performed with reference to the CEUS findings allowed sampling of the portion corresponding to the target-like heterogeneous enhancement pattern, confirming a benign schwannoma and supporting a conservative management strategy. Although the biopsy specimen showed loose Antoni B architecture without a definite Antoni A component, this finding does not necessarily contradict the CEUS findings. Histological re-evaluation demonstrated regional differences within the Antoni B component, including collagen-rich stroma in the outer to intermediate portions and more myxoid stroma in the central portion. These intratumoral differences in stromal composition and cellular density may have contributed to the heterogeneous enhancement pattern observed on CEUS. In addition, CEUS depicts intravascular perfusion rather than histological architecture itself. Moreover, the biopsy was performed under real-time B-mode US guidance after CEUS assessment rather than under simultaneous CEUS guidance. Therefore, exact spatial correspondence among the CEUS-enhancing area, the needle trajectory, and the histological field could not be established. Accordingly, the observed regional differences in the Antoni B component cannot be directly mapped to the CEUS enhancement pattern, and the proposed relationship should be interpreted cautiously. Future studies correlating CEUS findings with whole-specimen histopathological mapping may clarify whether specific CEUS enhancement patterns correspond to regional differences in stromal composition, vascular distribution, or Antoni A/B-predominant areas, and may further refine CEUS-informed targeted biopsy.

In Japan, CEUS has been investigated as a promising modality for breast imaging. Shima et al. demonstrated that CEUS delineates tumor extent more accurately than conventional B-mode US when compared with histopathological findings.^12)^ While previous work has focused primarily on extent delineation, our case suggests an additional role for CEUS in biopsy target selection based on intratumoral perfusion patterns. This CEUS-informed biopsy target selection approach may be particularly valuable in NF1 patients, in whom malignant transformation cannot be excluded a priori. This method may support individualized surgical decision-making, improve diagnostic confidence, and help avoid both under- and over-treatment of PNSTs presenting as breast lesions. In primary breast tumors, intratumoral heterogeneity in vascularity, necrosis, and viable tumor components may affect the diagnostic yield of biopsy specimens. CEUS can visualize real-time perfusion within a lesion and may help target viable or hypervascular regions, particularly when accurate pretreatment characterization is required before systemic therapy. Thus, CEUS-informed biopsy may also contribute to more precise tissue sampling in selected breast tumors. In patients with deep breast or retromammary lesions, particularly those with underlying NF1, CEUS-informed biopsy target selection may serve as a valuable adjunct in determining the necessity and extent of surgical intervention. This case highlights the importance of accurate preoperative localization of retromammary lesions and demonstrates that CEUS-informed biopsy target selection can support appropriate surgical decision-making while avoiding unnecessary surgical resection. In summary, accurate localization of deep breast lesions is crucial to avoid misdiagnosis and unnecessary surgical excision. Dynamic US evaluation combined with CEUS-informed biopsy target selection may improve diagnostic confidence in retromammary tumors. This approach may support appropriate clinical decision-making and help avoid unnecessary surgery, particularly in patients with underlying NF1.

## CONCLUSIONS

Dynamic US evaluation is useful for distinguishing retromammary lesions from true intramammary tumors. In addition, CEUS-informed biopsy target selection may improve diagnostic confidence and help avoid unnecessary surgical resection, particularly in patients with deep breast lesions or underlying NF1.

## SUPPLEMENTARY MATERIALS

Supplementary Video S1 Dynamic US demonstrating cranial displacement of the mass during right arm elevation. This mobility indicates that the lesion moves independently of the breast tissue, supporting its origin in the retromammary space and suggesting a neurogenic tumor.

Supplementary Video S2 After intravenous administration of a second-generation US contrast agent, the lesion shows a target-like heterogeneous enhancement pattern with relative central hypoenhancement and surrounding enhancement. The enhancement gradually fades but persists with slow retention in the late phase.

## DECLARATIONS

### Availability of data and materials

All data generated or analyzed during this study are included in the published article.
